# Enhanced proliferation of rabbit chondrocytes by using a well circulated nanoshock system

**DOI:** 10.1038/s41598-021-98929-2

**Published:** 2021-09-29

**Authors:** Sitansu Sekhar Nanda, Tuntun Wang, Hong Yeol Yoon, Seong Soo A. An, K. P. S. S. Hembram, Kwangmeyung Kim, Dong Kee Yi

**Affiliations:** 1 Department of Chemistry, Myongji University, Yongin, 03674 South Korea; 2 Center for Theragnosis, Biomedical Research Institute, Korea Institute of Science and Technology (KIST), Seoul, 02792 South Korea; 3 Department of Bionanotechnology, Gachon Medical Research Institute, Gachon University, Seongnam, 13120 South Korea; 4 Korea University (KU)-KIST Graduate School of Converging Science and Technology, Seoul, 02841 South Korea; 5 Optoelectronic Materials and Devices Research Center, Korea Institute of Science and Technology (KIST), Seoul, 02792 South Korea

**Keywords:** Biological techniques, Materials science, Nanoscience and technology

## Abstract

The gold nanorods (GNRs) embedded alginate-chitosan (scaffold), which was designed and fabricated to produce efficient handling of the cell proliferations. Scaffold embedded GNR (SGNR) and NIR (near infrared) irradiations are developing into an interesting medical prognosis tool for rabbit chondrocyte (RC) proliferation. SGNR contained a pattern of uniform pores. Biocompatibility and cellular proliferation achieved by disclosures to NIR irradiations, providing high cell survival. SGNR and NIR irradiations could produce mechanical and biochemical cues for regulating RCs proliferations. To determine the thermal stress, it exposed RCs to 39–42 °C for 0–240 min at the start point of the cell culture cycle. It produced photothermal stress in cellular surrounding (cells located adjacent to and within scaffold) and it deals with the proliferation behavior of RC. All the processes were modeled with experimental criteria and time evolution process. Our system could help the cell proliferation by generating heat for cells. Hence, the present strategy could be implemented for supporting cell therapeutics after transplantation. This implementation would open new design techniques for integrating the interfaces between NIR irradiated and non-irradiated tissues.

## Introduction

Stem cell injections to injury regions were assumed as an essential step for the renewal of tissues^1^. To maintain in the influential overhaul on tissues, stem cells must continue to restore under adverse conditions. One of the important factors could be the temperature, which could control many cellular processes. Alekseenko et al. observed the frequent occurrences of abrupt fluctuations of temperature^2^. They also detected the drastic increases in temperatures under many medical conditions, such as infections and contagious diseases^2^. More or less, the patients would produce fever (temperature higher 39 °C) as the relocation of stem cells progressed^3^. The cellular reactions to the heat production would be one of the best scrutinized cellular stress responses. Before and after transplantation, Heat shock protein (HSP) is an effective way to protect cells^4,5^. It can improve the therapeutic effect of bone marrow stem cells on a chemotherapy-induced premature ovarian insufficiency rat model^6^. Mild heating provides thermo-tolerance to cells from heat induced cellular damage^7,8^. HSP associated with bone, cartilage and mammalian osteoblast differentiation^9,10^. An elevated heating of 1.5–3 °C plays an important role in rat and dog bone growth stimulation^11^.

After the in vivo damages, the native diathermy could sponsor the reconstruction of the bone^12^. Local hyperthermia (38–41 °C) management could initiate the growth of the new bone in rats’ by distribution of heat. Mostly, radio waves, microwaves and ultrasound used to convey heat as local hyperthermia. In case of the musculoskeletal sicknesses, hyperthermia applied as a thermotherapy for recoveries of the disorder^12^. Hence, hyperthermia may affect the bone absorption and stimulate the native bone creation^13^. In addition, cartilage growth after the injury obtained by the microwave warming on the linkages of animal osteoarthritis^14^. The restricted temperature could also influence the direct heating of chondrocyte, osteoblasts, and osteoprogenitor cells, which were useful in recovering cells at the articular junctions. Initial reports of results showed that the mild heat (1.5–3 °C above regular body temperature) encouraged the advancements and in embryonic improvements of the bone in the animals^11,15^. They reported the mild increases from the native temperature at the sites of bone fractures, which could enhance the formation of new bone^16^.

Cell proliferation could be meliorated by translocations of proteins, ensuring correct folding and repairing denatured proteins^17^. Balancing the protein homeostasis was closely related with proinflammatory mechanisms, nitric oxide synthase activity and NF-κB signal pathways^18^. Heat shock proteins (HSPs) were over-expressed under stress conditions in cells, which could play a key role in the maintenance of protein homeostasis in cells through proper activations and the folding of client proteins^19–21^. Hence, controlling HSPs in cells may influence cell properties with correct conformations of proteins, helping cellular proliferations and differentiations.

**Figure 1:**
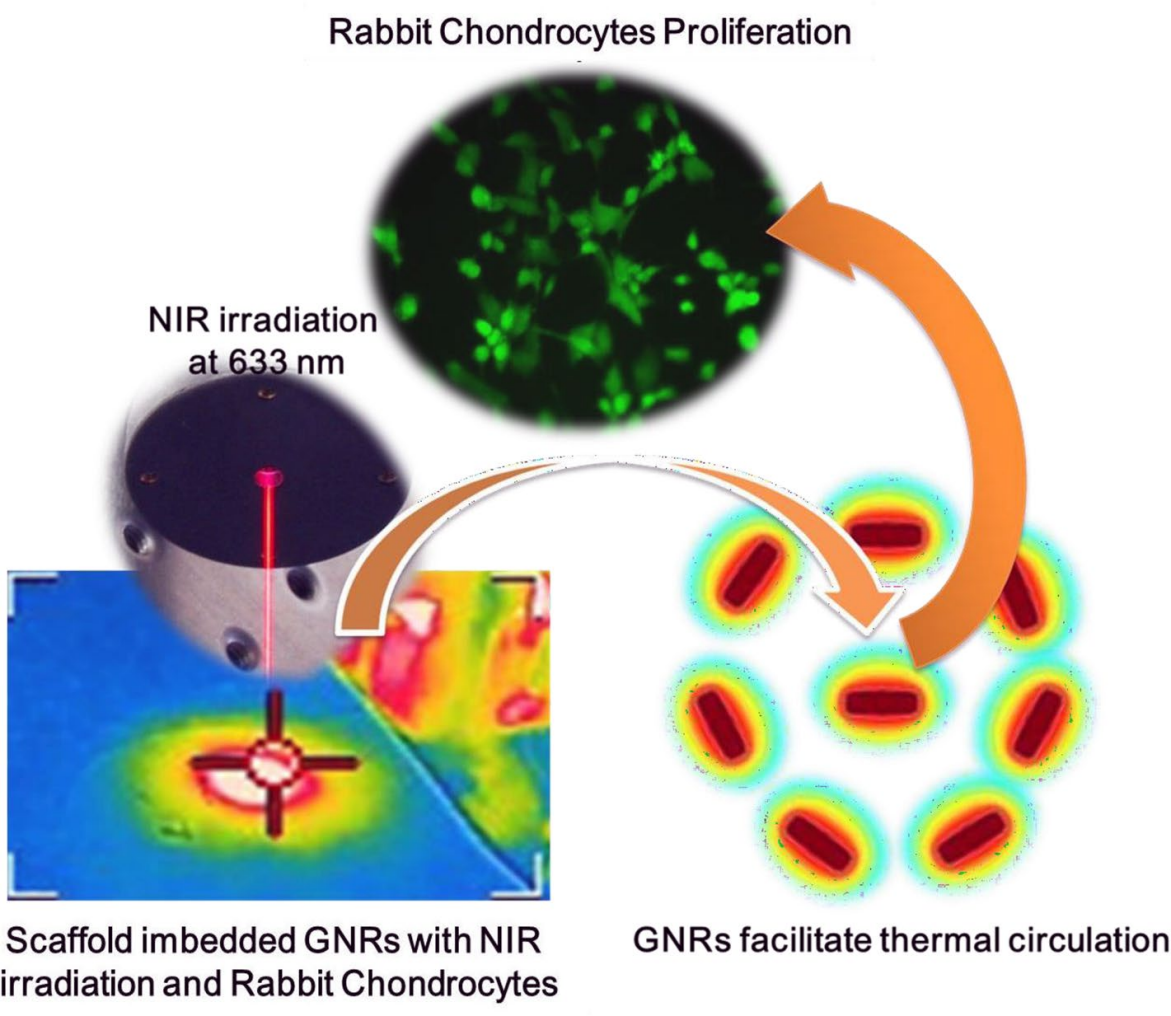
Schematic illustration of fabricating the biocompatible scaffold imbedded GNRs with NIR irradiation. Rabbit chondrocytes were seeded in scaffold imbedded GNRs with NIR irradiation at 633 nm laser. Rabbit chondrocyte proliferation occurred due to thermal circulation of GNRs.

Along with similar approaches, Chen et al.^22^ developed self-assembling hydrogel materials with peptides, which could generate the heat and control the cellular proliferations. For the cell-based therapies, various proteins were used to regenerate tissue, drug delivery, and cellular pathways for tissue renewals, including stem and immune cells^23^. In terms of the successful cellular therapy, it would be important to control the cellular proliferations and differentiations with enhanced capacity to alter the disease circumstances as described in Fig. 1.

In this study, we hypothesized that the circulatory nanoshock system may induce the heat shock proteins in cells, and the increased expressions of heat shock proteins could enhance the cellular proliferations. To address our hypothesis, SGNR was prepared by simple mixing methods. Scaffold showed in the multi pored network structure, which RCs could proliferate successfully for 21 days without in vitro toxicity.

## Materials and methods

### Materials

Rabbit chondrocytes were obtained from the New Zealand white rabbits. Cetyltrimethylammonium bromide (CTAB) was obtained from Alfa Aesar. Gold chloride trihydrate (HAuCl_4_∙3H_2_O), sodium borohydride (NaBH_4_), Silver nitrate (AgNO_3_), ascorbic acid, chitosan, calcein AM, MitoTracker and sodium alginate were purchased from Sigma-Aldrich. HSP 27, HSP 27 and HSP 90 were obtained from abcam.

### Instruments and methods

JEOL JSM-7500F with an acceleration voltage of 15 kV was used for field emission scanning electron microscopy (FE-SEM) study. JEOL 2000 FX microscopy was used for transmission electron microscopy (TEM) study with an acceleration voltage of 200 kV. An NIR laser with irradiation wavelength of 633 nm (MW-GX-980/3000 mW China) was used for the photothermal study. Varian instrument was used for the study of UV–Vis–spectroscopy.

### Isolation of chondrocyte from white rabbits

All experiments with animals were performed in compliance with the relevant laws and institutional guidelines of Institutional Animal Care and Use Committee (IACUC) in Korea Institute of Science and Technology (KIST), and IACUC approved the experiment (approved number of 2017-005). The animal study complies with ARRIVE guidelines.

Chondrocytes were isolated from the knee joint of New Zealand White rabbits (6-week-old, female; Orient, Korea)^24^. Briefly, the knee joint (articular cartilage) was chopped into small pieces to be digested in 0.05% type II collagenase containing DMEM–F12 (Dulbecco’s modified Eagle’s medium–F12). After 12 h of digestion, the digested tissue was filtrated through a 40 µm pore sized cell strainer (Fisher Scientific, Pittsburgh, PA). Phosphate buffered saline (PBS; pH 7.4) with 1% penicillin/streptomycin used for the wash and cell isolation, and they were maintained in DMEM–F12 supplemented with 10% (v/v) fetal bovine serum (FBS; Welgene) and 100 µg/mL streptomycin and 100 U/mL penicillin. The cells were cultured in a humidified 5% CO_2_ atmosphere at 37 °C, and the culture medium was changed every 3 days.

### RC culture technique

RC were suspended in chondrocyte culture medium (DMEM), and the number and viability of the RC were determined using a hemocytometer with trypan blue dye exclusion. The viability of isolated RC was over 95%. The freshly isolated RC were cultured in a petri dish in 5% CO_2_ atmosphere at 37 °C, grown to almost confluent and then passage by trypsin treatment. The chondrocyte at the third passage was used in this study.

The SGNR were immersed in the chondrocyte culture medium for 1 h prior to RC seeding. RC suspension (8 × 10^6^ cells/mL, 100 mL) was inoculated into the scaffold and incubated in a petri dish for 4 h RC attachment. During these 4 h, NIR irradiation was performed.

### Preparation of the GNR

Seed-mediated method was used for synthesis of GNR^26^. Briefly, 250 μL of 0.01M HAuCl_4_ was added in 7.5 mL of 0.1M CTAB, 600 μL of ice-cold 0.01M NaBH_4_ and the solution was kept under vigorous stirring condition at room temperature for preparation of seed solution. Separately, 400 μL of 0.01M HAuCl4, 64 μL of 0.1M ascorbic acid and 35.6 μL of 0.1 M AgNO_3_ were added in sequence to the 9.5 mL of 0.1M CTAB for preparation of growth solution. Then the seed solution (10 μL) was added to the growth solution, and mixed for 24 h at room temperature. Silver nitrate controlled GNR formations for better shape, and ascorbic acid served as a reducing agent.

### Scaffold synthesis

Alginate-chitosan-GNR slurry was prepared by addition of sodium alginate (1% w/v, 0.5 mL), chitosan (1% w/v, 0.5 mL) and GNR (43 µL, 0.125 mg/mL) in Deionized (DI) water. Then the solution was stirred for 4 h to get a uniform solution. Subsequently, the solution was kept at freeze (at − 10 °C for 24 h). Afterward, the solution was cross linked with calcium chloride (1%) for 15 min. The solution was washed properly with DI water.

### Center and edge temperature test of scaffold with GNR

SGNR was placed into a bottle and then add 1 mL media to hold the scaffold immersion in water. Test on the NIR laser which was placed at a fixed 10 cm distance away from the bottle to irradiate the scaffold. The laser power is 150 ± 2 mW. Using the thermocouple to test the scaffold center and edge temperature every 2 h for 24 h. All the experiment was repeated 3 times to ensure the accuracy.

### Flow cytometry analysis

FACScan cytometer and CellQuest Pro software were used for flow cytometry analysis. Isotonic PBS buffer pH 7.4 with cell suspension was gated on linear gains for light scattered channels, on a logarithmic scale for fluorescence channels with a minimum 10,000 cells for analysis in a single condition.

Harvesting of cells and aliquot up to (1 × 10^6^ cells) in a small petri dish was carried out. The cells were washed 2 times by adding 2 mL of PBS and centrifuging at 1200 rpm for 5 min, followed by decanting the buffer from the pelleted cells. The cells were resuspended in 100 µL of calcein AM staining buffer. To adjust flow cytometer settings for live, the live staining solution was added to a control tube of unstained cells. They were mixed and incubated for 15 min in the dark. Then it was rinsed with PBS two times. Then, 100 µL of Calcein AM staining solution was added to each sample just prior to live cell analysis. The point count of the viable cells was adjusted from a dot-plot of forward scatter versus calcein AM.

### Cell proliferation study

Scaffold labeling was assessed using the MTS assay. Cells after 1, 3, and 5 days of culture were washed with PBS. Cells were incubated with DMEM media containing 20% MTS solution for 4 h at 37 °C. The supernatant was collected and the absorbance values were measured at 490 nm (microplate reader). A blank sample containing no cells was subtracted from all measurements.

### Cell viability

Cell viability was assessed following nanoparticle labeling using Calcein AM viability staining and mitotracker assay. Calcein AM staining was performed by incubating the cells with a working solution of calcein AM (4 mm) for 45 minutes at 37 °C. The cells were rinsed with PBS and imaged using fluorescence microscopy (Zeiss DMI2000B microscope equipped with a Zeiss DFC 290 camera).

### Sulfated glycosaminoglycan (sGAG) quantitation

Sulfated Glycosaminoglycan (sGAG) Assay Kit was used for total sGAG quantitation. Briefly, 1,9-dimethyl-methylene blue dye (1 mL) reagent was added to 50 μL of the extract and kept for 30 min. The blue dye binds to sGAG and forms a purple dye–sGAG precipitate, which was separated from the unbound dye solution by centrifugation at 10,000×*g*. 200 μL of dissociation reagent was added to recover the sGAG-bound dye from the resulting pellet. Absorbance of chondroitin 4-sulfate standards, dye from sGAG samples, and blanks were quantified by using a microplate reader at 655 nm.

### Heat shock protein response

Staining of control, HSP 27, HSP 70 and HSP 90 in RC are done by Immunofluorescence. Briefly, cells were fixed in methanol for 30 minutes at − 20 °C, washed with PBS, and incubated in blocking solution (10% Bovine serum albumin in PBS) for 1 h at room temperature. Cells were stained with goat anti-rabbit IgG HRP-conjugated antibody diluted in blocking solution for 12 h at room temperature in humidified chambers. Cells were washed with PBS and then incubated with secondary antibody diluted 1/200 in blocking solution for 1 h at room temperature in opaque humidified chambers. The samples are analyzed by using fluorescence microscopy (530 nm wavelength). The standardized protocol was followed for control.

### Statistical analysis

The statistical analysis was carried out by Graph-Pad Prism (6.0) software using one-way ANOVA module. Different tests (e.g. Tukey test, Dunnett’s test) were performed for comparative mean and obtain P value. In our analysis the P values are less than 0.0001 in both the test which indicates better statistical significance. All experiments were repeated three times.

## Result and discussion

**Figure 2:**
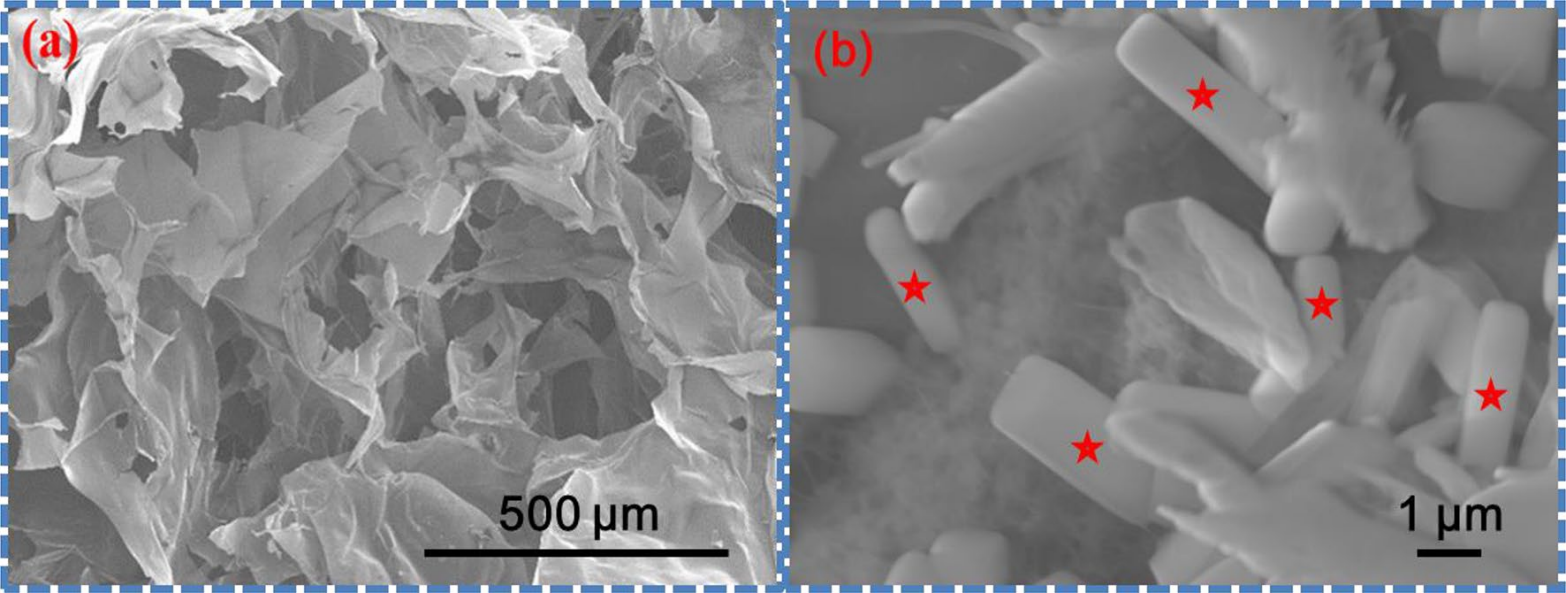
(**a**) Top view SEM image of SGNR, (**b**) Magnified image of SGNR clearly indicates GNRs (showed by astericks).

Scanning electron microscopy (SEM) images of SGNR were shown in Fig. 2a. The larger mats (length ~ 2–20 µm, height ~ 5 µm) are shown. These mats are lamella clusters of GNRs. Magnified images of SGNR (43 µL, 0.125 mg/mL) revealed high pore interconnectivity, randomly and densely packed in Fig. 2b, GNRs showed by asterisks.

Scaffold had a porous structure, ranging in sizes from 100 to 300 μm, which fitted into the appropriate size range for the growth of RC^25^. Better interconnectivity and mechanical strength observed with smaller pores^26^. Scaffold with larger pore are considered as weaker^27^. Infrared irradiations on GNR for increasing the temperatures were reported in the literature^28–34^.

**Figure 3:**
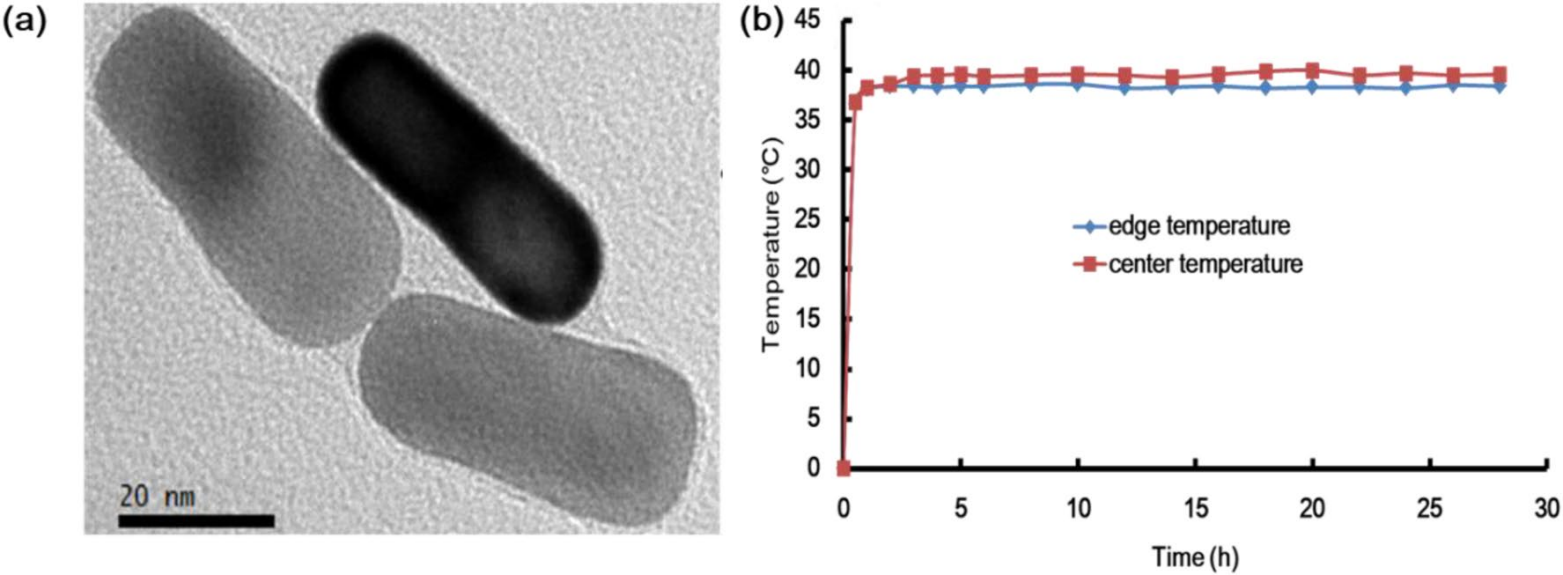
(**a**) TEM images of GNR. (**b**) Time vs temperature graph shown for edge temperature test and time vs temperature graph shown for center temperature test.

The morphology of GNR from TEM revealed a width and length below 50 nm, as noted in Fig. 3a. The effects of time vs temperature were investigated for the surface and center temperature tests, as shown in Fig. 3b. The temperature saturated at 38.2 °C. This drastic and fast saturation was a unique feature of our ECM system using Au rods as heat islands around cells.

**Figure 4:**
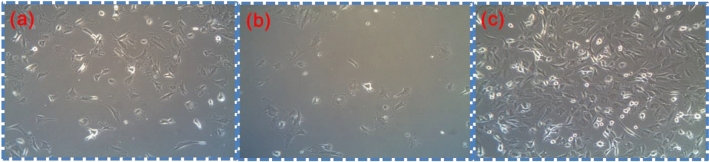
Optical image of rabbit chondrocyte (**a**) with scaffold, (**b**) immersed in SGNR, and (**c**) immersed in SGNR and treated with NIR irradiation.

The visualized morphology of RC under the inverted optical microscopy revealed differences between cells at the SGNR with and without NIR irradiations, as shown in Fig. 4. The results revealed the scaffolds were homogeneous to distribute (see Fig. 4a). With the addition of GNR, RC was mixed in the scaffold. GNR became heated faster than DMEM. Hence, the local temperature of the DMEM media was less than GNR. Next, the evaporation of DMEM took a place in the GNR’s proximity. Overall, multiple RC colonies formed on the plate after NIR irradiation, as shown in Fig. 4c, unlike without IR irradiations (Fig. 4b). The images obtained after 3 days of culture.

**Figure 5:**
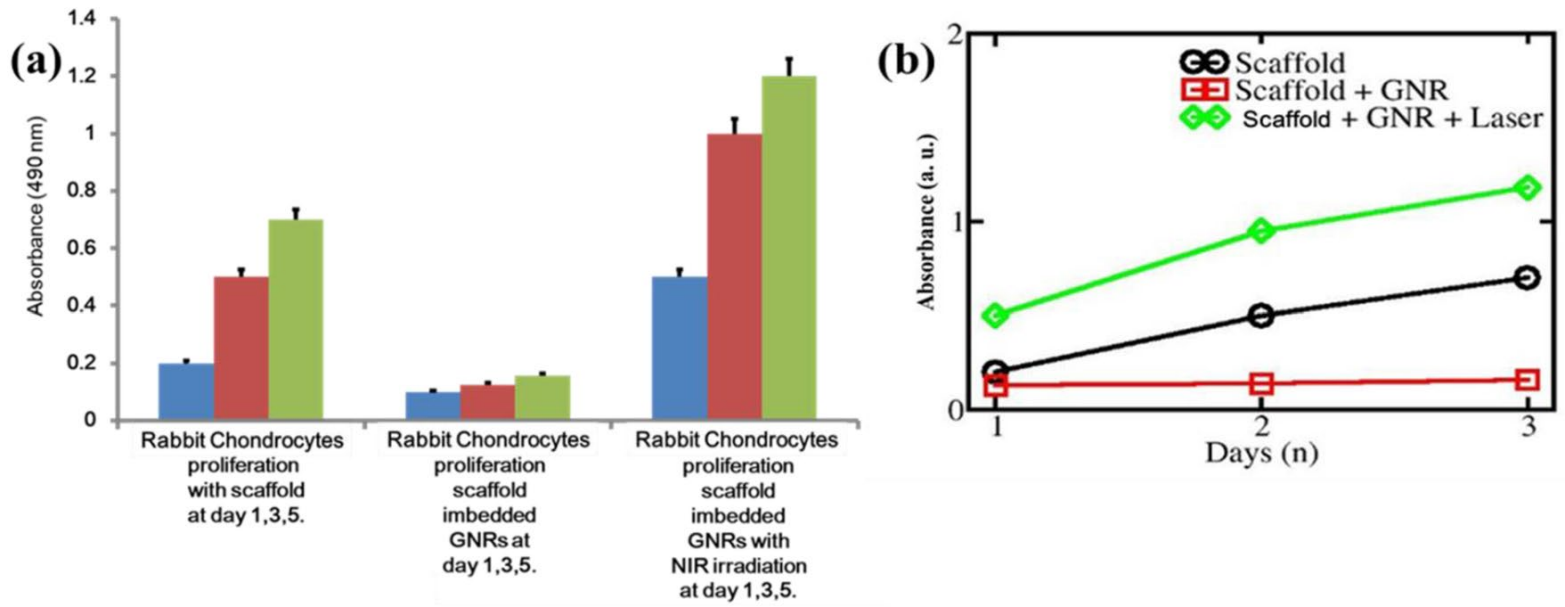
Above figure depicted as (**a**) Rabbit chondrocyte proliferation study with the scaffold (control). Rabbit chondrocytes proliferation study with SGNR. Rabbit chondrocyte immersed in SGNR were treated with 633 nm NIR irradiation at 180 mW for 4 h. Intensity profile (**b**) was monitored as a function of days.

RC was selected as the cell model for their morphological featured expressions and growth capability. RC was used to assess the cell proliferations on SGNR and without NIR irradiations. The only MTS assay was employed for the mapping of time, and the results were shown in Fig. 5. RC on both scaffolds (with and without NIR irradiations) showed the differences in the first day of cell culture, and their differences continued to increase over days. Significant differences in cell numbers were observed between the SGNR with and without NIR irradiations on day 1, day 3 and day 5 after cell culture. The RC numbers on the SGNR and NIR irradiation became higher than without the irradiation at day 1, day 3 and day 5, RC showed consistent viabilities in all locations of scaffold, and the NIR irradiation did not affect GNR contents. The flow cytometry analysis was conducted for their survival rate after day 7, day 14 and day 21. Cellular proliferation observed for the SGNR and NIR irradiation as compared with only SGNR. The uniform pore size of SGNR with NIR irradiation seemed to enable the transports of metabolic waste and various nutrients, as a result of RC viability. RC prefer differentiation into functional chondrocytes. This support RC as an source for cartilage regenerative therapy.

The expression of Beer–Lambert–Bouguer law is I = I_o_ e^−(*ϵcl*)^), where *ϵ* is the molar attenuation coefficient or absorptivity of the attenuating species, *c* is the concentration of the attenuating species, *l* is the optical path length, I is the observed intensity and I_o_ is the original intensity of the light. Basically, it is the relation of intensity with optical attenuation of physical material with certain concentration and the optical path length through the sample.

**Figure 6:**
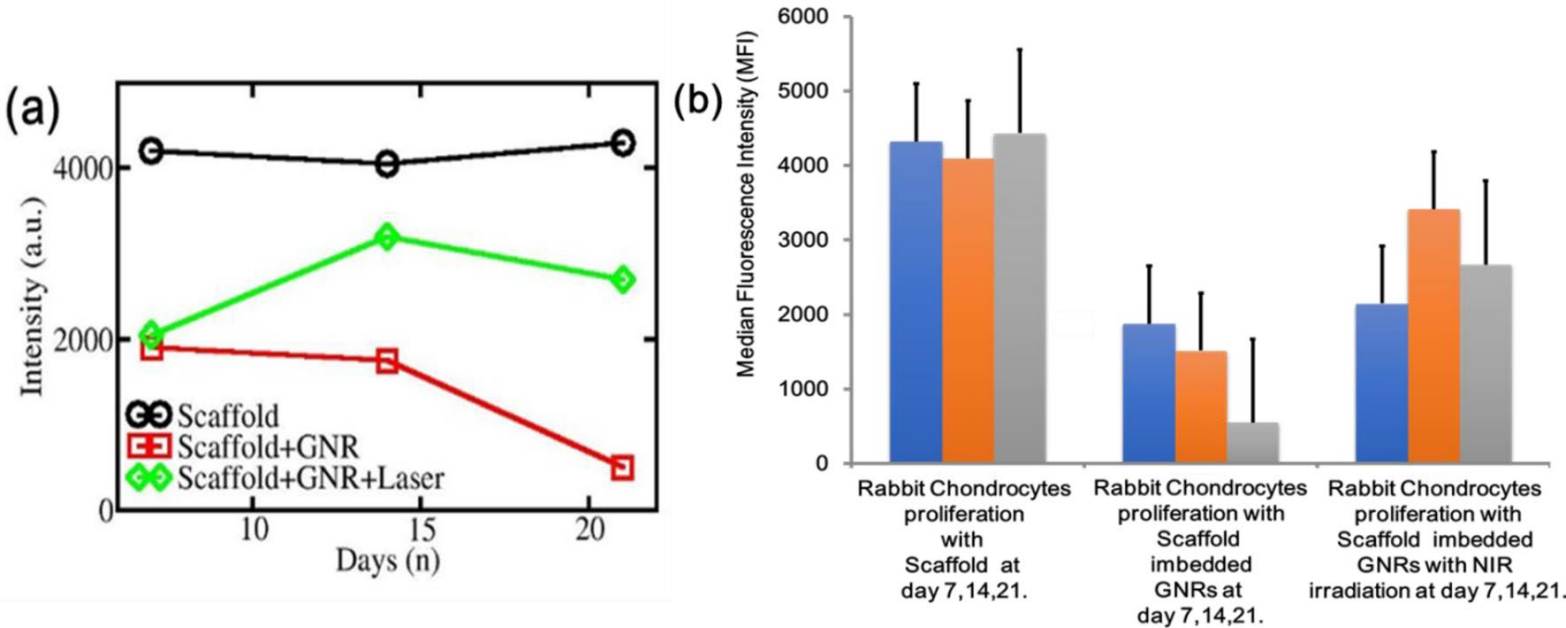
This above figure showed. (**a**) Intensity profile as a function of scaffold, SGNR, and SGNR with NIR irradiation. (**b**) Comparative flow cytometric histogram analysis of rabbit chondrocyte viability by using calcein-AM after 7, 14 and 21 days with specific conditions.

Although Beer–Lambert–Bouguer law could not be applied in the present case, due to immiscible/insoluble GNR in the scaffold, we tried to change the Beer–Lambert–Bouguer law with approximation. Hence, any change in the absorption intensity was related to the changes in these three parameters. Next, the absorption of SGNR can be expressed as (ϵ + µ) (c + δc) (l + δl). Again, the change in values i.e. µ, δc and δl, changed the intensity, as explained in Figs. 5a,b and 6a,b, respectively.

**Figure 7:**
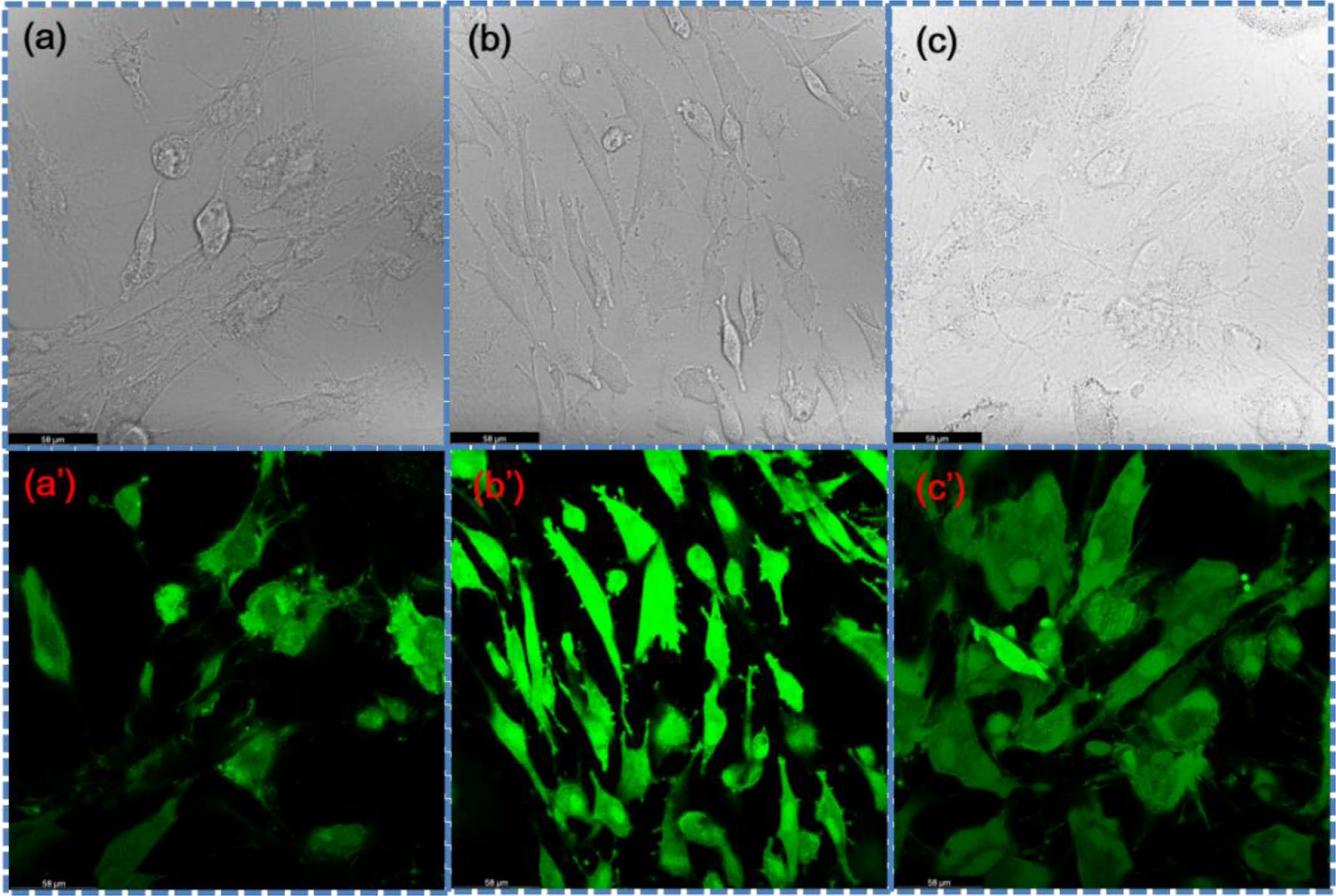
Confocal microscopic images of rabbit chondrocyte viability study staining (**a,aʹ**) with live assay kit with scaffold, (**b,bʹ**) with live assay kit immersed in SGNR, and (**c,cʹ**) with live assay kit immersed in SGNR and treated with NIR laser.

**Figure 8:**
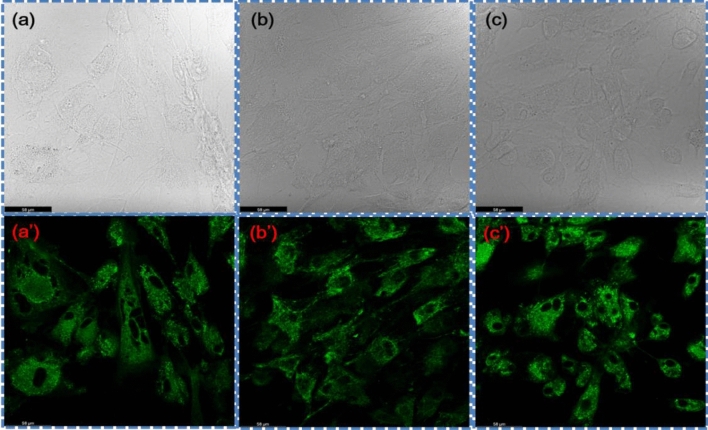
Confocal microscopic images of rabbit chondrocyte proliferation study staining (**a,aʹ**) with mitotracker assay kit with scaffold, (**b,bʹ**) with mitotracker assay kit immersed in SGNR, and (**c,cʹ**) with mitotracker assay kit immersed in SGNR and treated with NIR laser.

The confocal microscopy images of RC (1) with scaffolds, (2) SGNR, and (3) SGNR with NIR irradiation were shown in (Fig. 7a,aʹ,b,bʹ,c,cʹ), respectively. In both scaffold experiments, most RC were alive on SGNR with NIR irradiation. We found a few RCs in SGNR (Fig. 7b,bʹ). On SGNR, most RC formed isolated cells, exhibiting round-shape morphology. RC on SGNR with NIR irradiation formed fewer clusters than on the SGNR, exhibiting spread morphology. We observed excellent biocompatibility for SGNR with NIR irradiation. Mitotracker used for the study of the probable involvement of mitochondria in the pathways leading to proliferation (Fig. 8a,aʹ,b,bʹ,c,cʹ). The mitochondrial mass increased in case of SGNR with NIR irradiation, as shown in Fig. 8c,cʹ. Like the RC viability, mitochondrial change occurred.

RC proliferation, attachments and differentiation along the SGNR with NIR irradiation showed cellular compatibility and the suitability of novel techniques for tissue engineering applications. Our experimental results showed RC attached well to the SGNR with NIR irradiation for the enhanced cell proliferation techniques. SGNR and NIR irradiation techniques appeared to center on cellular proliferation property. The cell viability assay revealed the increased number of live cells on the SGNR after the NIR irradiation than without the irradiation (Fig. 5). These results showed our method promoted the cell expansion with excellent cell proliferation property.

To examine the impact of SGNR and NIR irradiation, RC viability was examined after 7, 14 and 21 days. We compared the viability of RC with scaffold, SGNR and SGNR with NIR irradiation. In case of embedded scaffold GNR, loss of esterase activity was observed.

The most typical size of GNR (40 nm and 10 nm in diameter) used for photothermal activation in the local surface area. Next, with assumption and initial approximation, the attributions form the NIR irradiation would affect the interface of GNR and scaffold. The effective surface area *A* = *n*×2*πrh* (*r* + *h*), where *n* is the number of GNR, *r* and *h* are the average radius and height of the GNR. Hence, the molar attenuation coefficient should have been changed by the *ϵ* + *δϵ* and *µ* + *δµ*, *where δϵ* and *δµ* are the contribution form scaffold and GNR, respectively. *δϵ* and *δµ* are proportional to the effective area. In our experimental procedures, the parametric values were placed to justify the equation, although the effect of other many parameters could not be neglected. Since no targeting agent was used to promote GNR uptake, the results would show the effects of uptake by RC were responsible for the NIR irradiation of GNR. GNR aggregated when suspended in cell culture medium, as clear from the change in temperature in Fig. S1a–s. Macroscopic image of scaffold and SGNR presented in Fig. S1t,u. GNR aggregation could most likely be attributed to the heat response on the surface of the SGNR. It could be possible that the heat response could promote the uptake of SGNR by the Rabbit chondrocyte. There would be several endocytosis pathways and effects of this HS technique, as shown in Fig. S1. The vibrational population (normally, the shock excited phonons and molecular vibrations) could have been relaxed because of the thermal distribution^35–37^. A fast cycle of heating and cooling (quenching) was demonstrated in Fig. S1.

By heating RC revealed a threshold of thermal dose for maximum HSP expression. HSP protocol identified 240 min from this study for an excellent inspection time. So, heating purposes with higher thermal increase rates (such as by laser) should be corrected to determine whether it could affect the heating rate on HSP expressions. Interpretation of the correlation between the HSP 27 (Fig. S2a,aʹ), HSP70 (Fig. S2b,bʹ) and HSP 90 (Fig. S2c,cʹ) expressions demonstrated. One possible simple statement was that the HSP response mounted by RC was classified and susceptible. The variant of the HS response was that it expressed an attempt by the RC to enhance its effectiveness to eliminate misfolded HSP. This stand was confirmed because over expressing HSP 27, HSP 70 and HSP 90 could suppress the aggregation of HSP, as illustrated in Fig. S2, the HSP response may suggest that HSP was penetrated the reasonable efficiency and supported in proliferation.

However, it was necessary to investigate how RC reacted to the use of biomaterials. Bone formation, followed by the cellular hierarchy, initiated the differentiation of RC from osteoprogenitor cells and then preosteoblasts and osteoblasts^38^. Our results supported the above suggestions, where RC expressions were altered by culture temperature and systems^39^. Our study revealed few limitations. The effects of culture temperature on protein levels and the effect of culture temperature on RC metabolism were remaining to be investigated. Only one cell line from one individual was analyzed. Therefore, in order to generalize our findings, larger studies will be performed.

**Figure 9:**
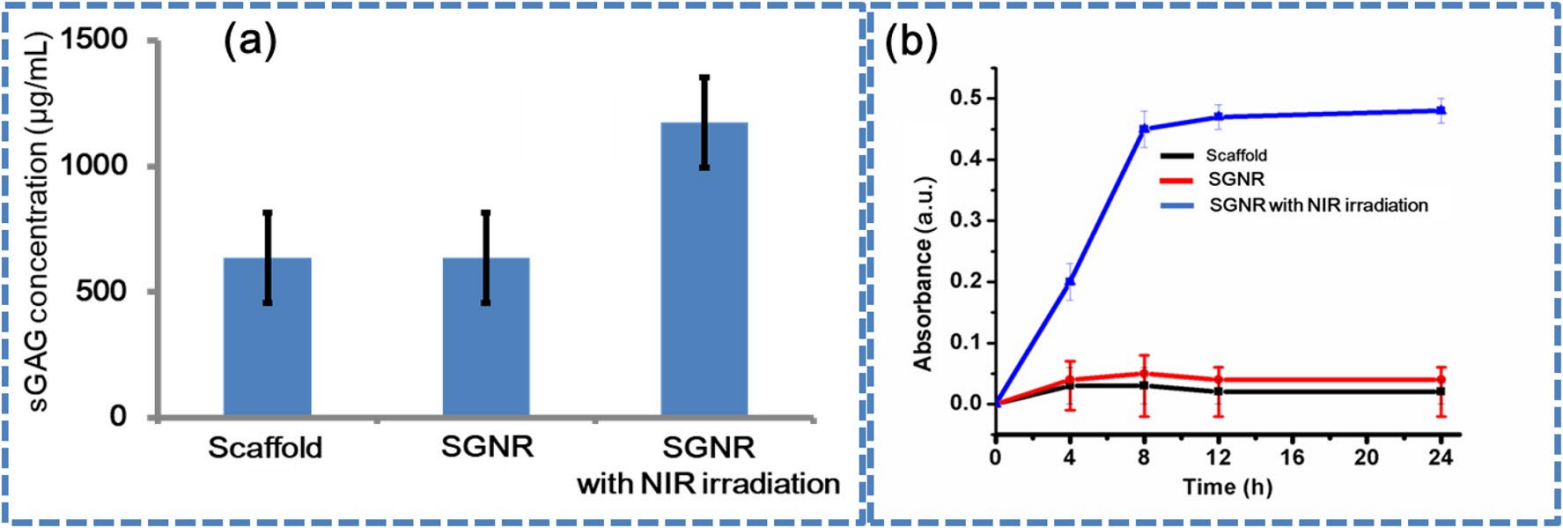
(**a**) sGAG quantitation of rabbit chondrocyte control, with scaffold, immersed in SGNR, and immersed in SGNR and treated with NIR irradiation. (**b**) Absorption profile of BSA at different time with scaffolds, SGNR and SGNR with NIR irradiation.

The regulation of different cellular behaviors, such as proliferation, adhesion, differentiation, could be influenced by protein affinity and scaffold surface^40,41^. As expected, shown in Fig. 9a, following a 5-day culture, SGNR with NIR irradiation, showed a significant increase in the GAG content. sGAG accumulation was greater in SGNR with NIR irradiation compared with SGNR and scaffold. Variation in extracellular matrix synthesis and cellular proliferation handled this dissimilar level of sGAG accumulation. Here, temperature was acting as gradients in nutrients and SGNR was functioning as regulatory molecules within the system. It was reported that heat stimulus is responsible for increase in viability of chondrocytes^42^. Duration of heat exposure affects metabolism of chondrocyte. Heat stimulus reduce the apoptosis of chondrocytes, through the induction of HSPs^43^. In order to evaluate the protein interactions of scaffolds embedded without and with GNR, BSA was used as a model protein. Higher adsorption levels of the BSA (765%) on SGNR and NIR irradiation were observed higher than without the NIR irradiation after 24 h. This adsorption capacity of BSA became saturated after 12 h, as shown in Fig. 9b. Higher concentrations of BSA were adsorbed on the SGNR after NIR irradiation and improved the protein affinity substrates. Other studies found that electrostatic interactions of nanomaterials handled the increased adsorptions of DNA^44^, proteins^45^, and small molecule drugs^46^. Scaffold surface deposition of nutritious components was suitable for cell survival and proliferations. This method promotes the RC adhesions and growth on SGNR and NIR irradiation.

## Conclusions

In conclusion, it developed an alternative method for fabrication of SGNR and NIR irradiation. NIR irradiations of the SGNR could reach the temperature of 39–42 °C. This temperature could facilitate to produce the photothermal stress in the cellular surroundings. The results of the responses of RC towards the SGNR and NIR irradiations would be the first report of such scaffold. We did not document this method for other cell types. It did not identify the response profiles of RC to such a thermal stress until the present finding. Thus, this study offered a strategy to define the dynamics of thermal inductions of RC in vitro.

## Supplementary Information

Supplementary Information.

## References

[1] Cmielova J (2012). Gamma radiation induces senescence in human adult mesenchymal stem cells from bone marrow and periodontal ligaments. Int. J. Radiat. Biol..

[2] Alekseenko LL (2012). Heat shock induces apoptosis in human embryonic stem cells but a premature senescence phenotype in their differentiated progeny. Cell Cycle.

[3] Spitzer TR (2001). Engraftment syndrome following hematopoietic stem cell transplantation. Bone Marrow Transplant..

[4] Sart S, Ma T, Li Y (2014). Preconditioning stem cells for in vivo delivery. BioResearch Open Access.

[5] Choudhery MS, Badowski M, Muise A, Harris DT (2015). Effect of mild heat stress on the proliferative and differentiative ability of human mesenchymal stromal cells. Cytotherapy.

[6] Ding C (2018). Human amniotic mesenchymal stem cells improve ovarian function in natural aging through secreting hepatocyte growth factor and epidermal growth factor. Stem Cell Res. Ther..

[7] Landry J, Bernier D, Chrétien P, Nicole LM, Tanguay RM, Marceau N (1982). Synthesis and degradation of heat shock proteins during development and decay of thermotolerance. Can. Res..

[8] Li GC (1985). Elevated levels of 70,000 dalton heat shock protein in transiently thermotolerant Chinese hamster fibroblasts and in their stable heat resistant variants. Int. J. Radiat. Oncol. Biol. Phys..

[9] Loones MT, Morange M (1998). Hsp and chaperone distribution during endochondral bone development in mouse embryo. Cell Stress Chaperones.

[10] Shakoori AR (1992). Expression of heat shock genes during differentiation of mammalian osteoblasts and promyelocytic leukemia cells. J. Cell. Biochem..

[11] Richards V, Stofer R (1959). The stimulation of bone growth by internal heating. Surgery.

[12] Ogawa H (1990). Effects of the localized thermal enhancement on new bone formation following mechanical expansion of the rat sagittal suture. J. Jpn. Orthod. Soc..

[13] Brookes M, Richards DJ, Singh M (1970). Vascular sequelae of experimental osteotomy. Angiology.

[14] Takahashi KA (2009). Hyperthermia for the treatment of articular cartilage with osteoarthritis. Int. J. Hyperth..

[15] Doyle JR, Smart BW (1963). Stimulation of bone growth by short-wave diathermy. JBJS.

[16] Brodin H (1955). Longitudinal bone growth, the nutrition of the epiphyseal cartilages and the local blood supply: An experimental study in the rabbit. Acta Orthop. Scand..

[17] Buchner J (1996). Supervising the fold: Functional principles of molecular chaperones. FASEB J..

[18] Wong HR (1998). Potential protective role of the heat shock response in sepsis. New Horizons (Baltimore).

[19] Kim YE, Hipp MS, Bracher A, Hayer-Hartl M, Ulrich Hartl F (2013). Molecular chaperone functions in protein folding and proteostasis. Annu. Rev. Biochem..

[20] Saibil H (2013). Chaperone machines for protein folding, unfolding and disaggregation. Nat. Rev. Mol. Cell Biol..

[21] Hartl FU, Bracher A, Hayer-Hartl M (2011). Molecular chaperones in protein folding and proteostasis. Nature.

[22] Chen J, Shi ZD, Ji X, Morales J, Zhang J, Kaur N, Wang S (2013). Enhanced osteogenesis of human mesenchymal stem cells by periodic heat shock in self-assembling peptide hydrogel. Tissue Eng. A.

[23] Green EM, Lee RT (2013). Proteins and small molecules for cellular regenerative medicine. Physiol. Rev..

[24] Yoon HI (2016). Bioorthogonal copper free click chemistry for labeling and tracking of chondrocytes in vivo. Bioconjug. Chem..

[25] Li Z, Ramay HR, Hauch KD, Xiao D, Zhang M (2005). Chitosan–alginate hybrid scaffolds for bone tissue engineering. Biomaterials.

[26] Felfel RM, Gideon-Adeniyi MJ, Hossain KMZ, Roberts GA, Grant DM (2019). Structural, mechanical and swelling characteristics of 3D scaffolds from chitosan-agarose blends. Carbohyd. Polym..

[27] Loh QL, Choong C (2013). Three-dimensional scaffolds for tissue engineering applications: Role of porosity and pore size. Tissue Eng. B Rev..

[28] Ramasamy M, Kim S, Lee SS, Yi DK (2016). Recyclable photo-thermal nano-aggregates of magnetic nanoparticle conjugated gold nanorods for effective pathogenic bacteria lysis. J. Nanosci. Nanotechnol..

[29] Zhu Y, Ramasamy M, Yi DK (2014). Antibacterial activity of ordered gold nanorod arrays. ACS Appl. Mater. Interfaces.

[30] Mallick S, Sun IC, Kim K, Yi DK (2013). Silica coated gold nanorods for imaging and photo-thermal therapy of cancer cells. J. Nanosci. Nanotechnol..

[31] Ramasamy M, Lee SS, Yi DK, Kim K (2014). Magnetic, optical gold nanorods for recyclable photothermal ablation of bacteria. J. Mater. Chem. B.

[32] Yi DK (2011). A study of optothermal and cytotoxic properties of silica coated Au nanorods. Mater. Lett..

[33] Ramasamy M, Zhu Y, Paik U, Yi DK (2014). Synthesis and anti-bacterial activity of AuNRs–PS–MNPs. Mater. Lett..

[34] Yi DK (2010). Matrix metalloproteinase sensitive gold nanorod for simultaneous bioimaging and photothermal therapy of cancer. Bioconjug. Chem..

[35] Dlott DD, Fayer MD (1990). Shocked molecular solids: Vibrational up pumping, defect hot spot formation, and the onset of chemistry. J. Chem. Phys..

[36] Tokmakoff A, Fayer MD, Dlott DD (1993). Chemical reaction initiation and hot-spot formation in shocked energetic molecular materials. J. Phys. Chem..

[37] Tas G, Franken J, Hambir SA, Hare DE, Dlott DD (1997). Ultrafast Raman spectroscopy of shock fronts in molecular solids. Phys. Rev. Lett..

[38] Long MW (2001). Osteogenesis and bone-marrow-derived cells. Blood Cells Mol. Dis..

[39] Foldager CB, Nielsen AB, Munir S, Ulrich-Vinther M, Søballe K, Bünger C, Lind M (2011). Combined 3D and hypoxic culture improves cartilage-specific gene expression in human chondrocytes. Acta Orthop..

[40] Alves NM, Pashkuleva I, Reis RL, Mano JF (2010). Controlling cell behavior through the design of polymer surfaces. Small.

[41] Woo KM, Seo J, Zhang R, Ma PX (2007). Suppression of apoptosis by enhanced protein adsorption on polymer/hydroxyapatite composite scaffolds. Biomaterials.

[42] Hojo T, Fujioka M, Otsuka G, Inoue S, Kim U, Kubo T (2003). Effect of heat stimulation on viability and proteoglycan metabolism of cultured chondrocytes: Preliminary report. J. Orthop. Sci..

[43] Ito A, Aoyama T, Tajino J, Nagai M, Yamaguchi S, Iijima H, Zhang X, Akiyama H, Kuroki H (2014). Effects of the thermal environment on articular chondrocyte metabolism: A fundamental study to facilitate establishment of an effective thermotherapy for osteoarthritis. J. Jpn. Phys. Ther. Assoc..

[44] Wu M, Kempaiah R, Huang PJJ, Maheshwari V, Liu J (2011). Adsorption and desorption of DNA on graphene oxide studied by fluorescently labeled oligonucleotides. Langmuir.

[45] Hu W (2011). Protein corona-mediated mitigation of cytotoxicity of graphene oxide. ACS Nano.

[46] Goenka S, Sant V, Sant S (2014). Graphene-based nanomaterials for drug delivery and tissue engineering. J. Control. Release.

