# Supplementary material for: Enhanced proliferation of rabbit chondrocytes by using a well circulated nanoshock system
Source: Sci Rep. 2021 Sep 29;11:19388. doi: 10.1038/s41598-021-98929-2 (PMC8481538; doi:10.1038/s41598-021-98929-2)
Supplement: Supplementary file 1 — Supplementary Information. [file 41598_2021_98929_MOESM1_ESM.docx]

***Supporting Information for***

**Enhanced proliferation of rabbit chondrocytes by using a well circulated nanoshock system**

Sitansu Sekhar Nanda,^a^ Tuntun Wang,^a^ Hong Yeol Yoon,^b^ Seong Soo A. An,^c^ K. P. S. S. Hembram,^e^ Kwangmeyung Kim,^bd*^ Dong Kee Yi^a*^

^a^Department of Chemistry, Myongji University, Yongin, 03674, South Korea

^b^ Center for Theragnosis, Biomedical Research Institute, Korea Institute of Science and Technology (KIST), Seoul, 02792, South Korea.

^c^ Department of Bionanotechnology, Gachon Medical Research Institute, Gachon University, Seongnam, 13120, Republic of Korea

^d^ Korea University (KU)-KIST Graduate School of Converging Science and Technology, Seoul, 02841,South Korea

^e^Optoelectronic Materials and Devices Research Center, Korea Institute of Science and Technology (KIST), Seoul, 02792, South Korea

*Corresponding author:

Email: [vitalis@mju.ac.kr](mailto:vitalis@mju.ac.kr); kim@kist.re.kr


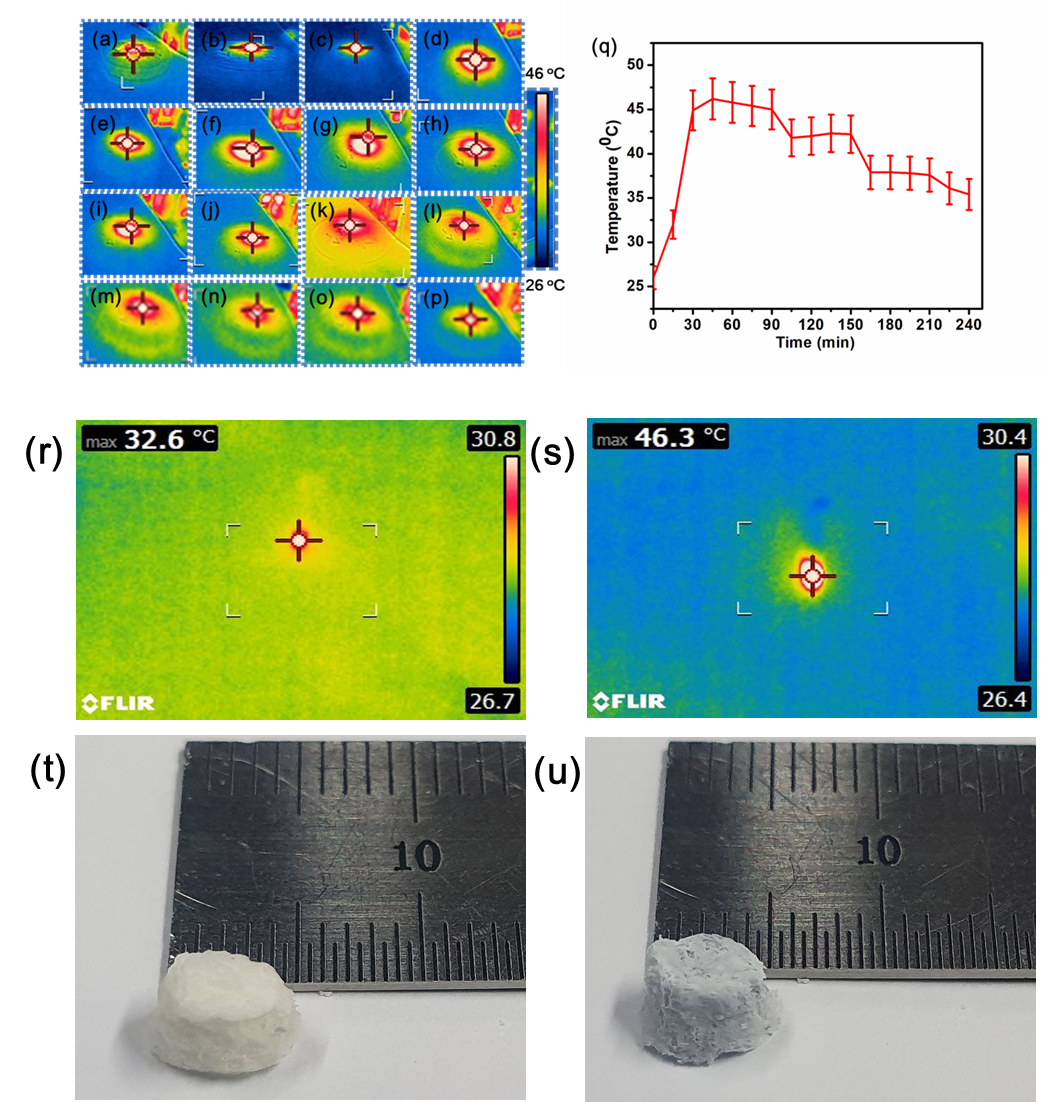


**Figure S1** (a) Image was taken after 15 min (a), 30 min (b), 45 min (c), 60 min (d), 75 min (e), 90 min (f), 105 min (g), 120 min (h), 135 min (i), 150 min (j), 165 min (k), 180 min (l), 195 min (m), 210 min (n), 225 min (o), 240 min (p) laser irradiation and temperature was 32.0 ^°^C, 44.9 ^°^C, 46.2 ^°^C, 45.8^°^C, 45.4 ^°^C, 45.0 ^°^C, 41.8 ^°^C, 42.0 ^°^C, 42.3 ^°^C, 42.2 ^°^C, 37.9 ^°^C, 37.9 ^°^C, 37.8 ^°^C, 37.6 ^°^C, 36.1 ^°^C, 35.4 ^°^C respectively. (q) Quantified data (a-p) presented. (r) Thermal images of SGNR (s) Thermal images of SGNR with NIR irradiation. (t) Macroscopic image of scaffold (u) Macroscopic photograph of scaffold imbedded GNR.


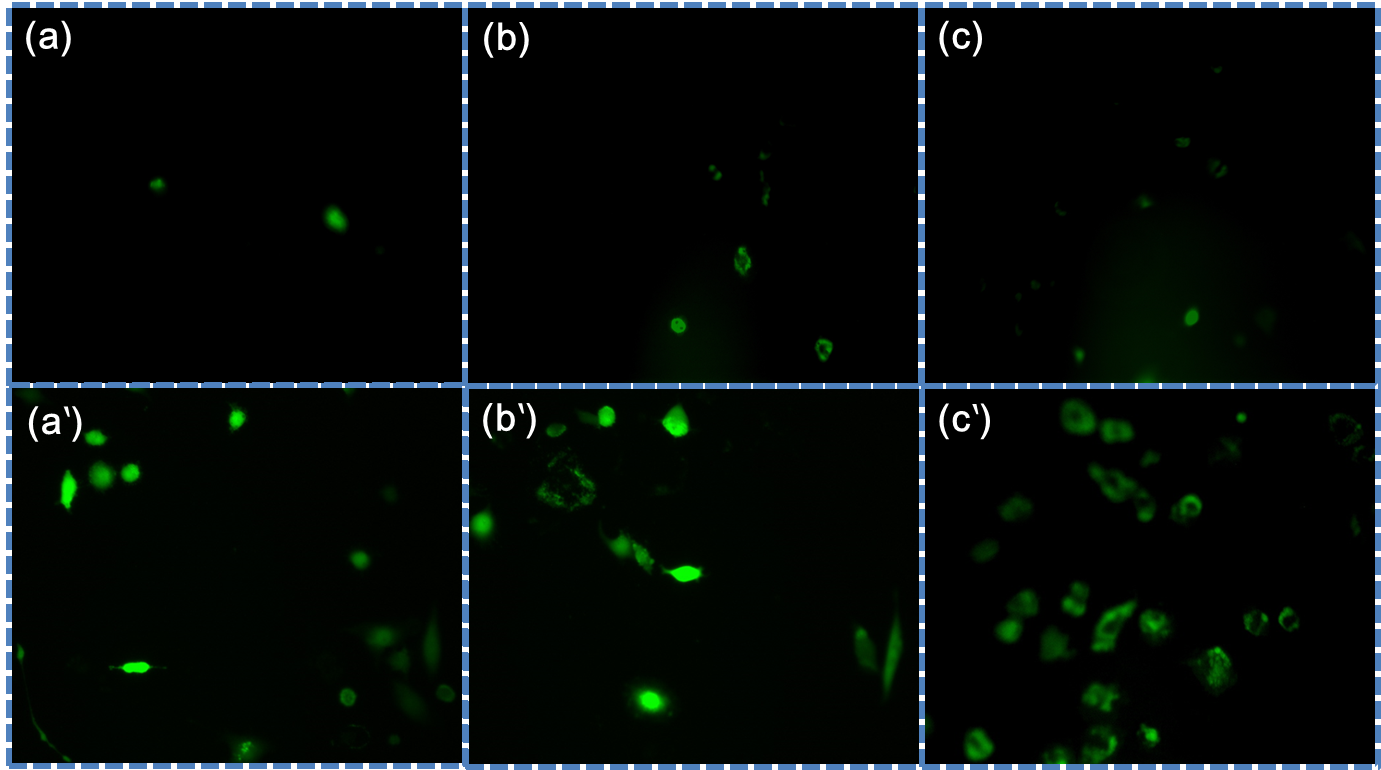


**Figure S2** (a) HSP 27 response for Rabbit Chondrocytes immersed in scaffold imbedded with GNRs and treated with 633 nm laser irradiation at 180 mW for 4 hours. (b) HSP 70 response for Rabbit Chondrocytes immersed in scaffold imbedded GNRs and treated with 633 nm laser irradiation at 180 mW for 4 hours. (c) HSP 90 response for Rabbit Chondrocytes immersed in scaffold imbedded with GNRs and treated with 633 nm laser irradiation at 180 mW for 4 hours.
